# The Advantages of Atmospheric Distension of the Rectum, with Dislodgement of Small Intestines

**Published:** 1894-12

**Authors:** 


THE ADVANTAGE OF ATMOSPHERIC DISTENSION OF THE RECTUM, WITH DISLODGEMENT OF THE SMALL INTESTINES, IN THE BIMANUAL EXAMINATION OF UTERUS, OVARIES, AND TUBES.—Howard A. Kelly (*American Journal of Obstetrics*, May, 1894) suggests a procedure for overcoming the embarrassment experienced by the crowding of the small intestines down into the pelvis and the consequent liability of making a false diagnosis of pelvic disease. Coils of small intestines in the pelvis containing fluid often feel tense and fluctuating, and thus readily impose themselves upon the examiner as large cystic ovaries, or leave him in doubt as to their true nature. The complete removal of these impediments may be satisfactorily effected in the following manner: The patient is placed in the knee-breast posture, with shoulders on the table and hips high and thighs vertical. The anal orifice is opened by a small speculum or tube, allowing the air to rush into the rectum. The explanation of this phenomenon is that, upon assuming the knee-breast posture, the small intestines gravitate along the anterior abdominal wall into the upper abdomen towards the diaphragm, creating a suction at the most elevated portion, which is the pelvic extremity, by means of which the whole ampulla and rectum balloon out with air as soon as the anus is opened, and the distended rectum applies itself broadly over the posterior surface of the uterus and left broad ligament. Before making such an examination, both rectum and bladder must be thoroughly emptied. Immediately after filling the rectum with air the tube is removed, the patient placed in the ordinary dorsal position with limbs flexed upon the abdomen, and the bimanual examination made per rectum and abdomen. The index finger introduced within the anus experiences at once the sensation of entering a large cavity filled with air, in which the customary resistance is absent. The communication with the upper bowel between the utero-sacral folds is, under these circumstances, readily found, and the finger is conducted behind the broad ligament, when, on using the outside hand in assistance, uterus, broad ligaments, ovaries, and tubes are at once palpated directly through the rectal wall, without resistance and with startling distinctness. The true pelvic viscera thus seen, as it were, to be skeletonized in the pelvis, lying so clearly exposed to touch that the minuter surface peculiarities, fissures, and elevations, and changes in consistence, can be detected, and a diagnosis made more satisfactorily, more rapidly, and with far less effort than under ordinary conditions.—*University Medical Magazine.*

